# Preparative semiconductor photoredox catalysis: An emerging theme in organic synthesis

**DOI:** 10.3762/bjoc.11.173

**Published:** 2015-09-09

**Authors:** David W Manley, John C Walton

**Affiliations:** 1 University of St. Andrews, EaStCHEM School of Chemistry, St. Andrews, Fife, KY16 9ST, UK

**Keywords:** carboxylic acids, free radicals, organic synthesis, photocatalysis, titania

## Abstract

Heterogeneous semiconductor photoredox catalysis (SCPC), particularly with TiO_2_, is evolving to provide radically new synthetic applications. In this review we describe how photoactivated SCPCs can either (i) interact with a precursor that donates an electron to the semiconductor thus generating a radical cation; or (ii) interact with an acceptor precursor that picks up an electron with production of a radical anion. The radical cations of appropriate donors convert to neutral radicals usually by loss of a proton. The most efficient donors for synthetic purposes contain adjacent functional groups such that the neutral radicals are resonance stabilized. Thus, ET from allylic alkenes and enol ethers generated allyl type radicals that reacted with 1,2-diazine or imine co-reactants to yield functionalized hydrazones or benzylanilines. SCPC with tertiary amines enabled electron-deficient alkenes to be alkylated and furoquinolinones to be accessed. Primary amines on their own led to self-reactions involving C–N coupling and, with terminal diamines, cyclic amines were produced. Carboxylic acids were particularly fruitful affording C-centered radicals that alkylated alkenes and took part in tandem addition cyclizations producing chromenopyrroles; decarboxylative homo-dimerizations were also observed. Acceptors initially yielding radical anions included nitroaromatics and aromatic iodides. The latter led to hydrodehalogenations and cyclizations with suitable precursors. Reductive SCPC also enabled electron-deficient alkenes and aromatic aldehydes to be hydrogenated without the need for hydrogen gas.

## Introduction

Synthetic chemistry is the springboard into innovative new products and materials for improving the wellbeing of society. In this context discovering cleaner, greener, more environmentally friendly preparative chemical methods has grown to an enterprise of great consequence. Utilizing energy from the Sun, rather than burning fossil fuels or employing corrosive acids or bases, is an attractive ‘green’ prospect. To accomplish chemical transformations it is necessary to make and break chemical bonds. However, about 98% of the energy reaching Earth’s surface from the Sun is in fact in the IR, visible and UVA regions, at wavelengths matching the energy of only a few particularly weak bonds. The result is that unappealing initiators such as peroxides or azo compounds normally have to be employed. Two different classes of materials, collectively known as photoredox catalysts (PRCs), have been exploited that enable visible and UVA radiation to be put to use without directly breaking chemical bonds. Typically, PRCs when photoexcited mediate electron transfer between suitable precursor substrates thus generating radical ions and thereby launching fresh, uncommon reaction sequences.

Homogeneous PRCs form one class that encompasses soluble organic dyes [1] as well as transition metal complexes; particularly those of Ru and Ir. Study of the latter markedly escalated from 2008 thanks to papers from the groups of MacMillan [2], Yoon [3] and Stephenson [4]. The popularity of homogeneous PRCs is due to their ease of synthesis and stability as well as their excellent photoredox properties. Furthermore, they can be activated by visible light, precluding the requirement for specialized irradiation setups, and their reactivity can be tuned by altering the substitution pattern on the ligands and by changing the metal. In the case of the prototypical PRC, Ru(bpy)_3_^2+^, absorption of a photon generates the long lived triplet species *Ru(bpy)_3_^2+^ that can act both as a reductant and an oxidant. Electron transfer to an acceptor molecule A generates the A^–•^ radical anion. Alternatively, *Ru(bpy)_3_^2+^ acts as an oxidant by accepting an electron from a suitable donor molecule D thus creating the radical cation D^+•^. Successful protocols have been developed for a variety of preparations including: enantioselective α-alkylations of aldehydes with radicals derived from α-bromocarbonyl components, decarboxylative arylations of amino acids, diastereoselective preparations of *cis*-cyclobutanes via [2 + 2] cycloadditions of enones, selective reductions of benzylic and α-carbonyl halides and, with *fac*-Ir(*ppy*)_3_, reductions of unactivated alkyl iodides [5–8].

Furthermore, the value of homogeneous PRC has been showcased by its exploitation in a number of total syntheses. For example, (+)-gliocladin C was synthesized by Stephenson [9], natural product heitziamide A was made by Yoon and co-workers via a PRC Diels−Alder cycloaddition [10], and a Ru(bpy)_3_^2+^ reaction with an *N*-(acyloxyl)phthalimide was employed by the Overman group in their synthesis of (−)-aplyviolene [11].

**Figure 1:**
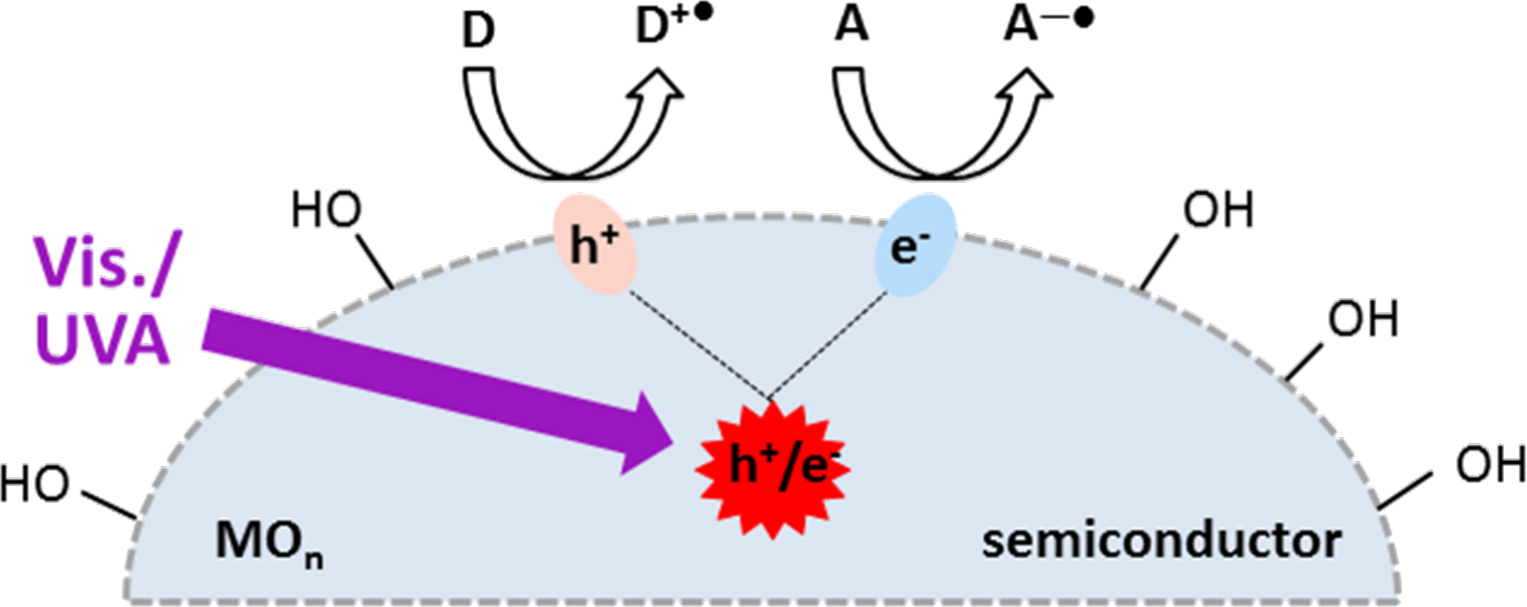
Production and utilization of h^+^ and e^–^ by photoactivation of a semiconductor.

Heterogeneous PRCs form a second class consisting of semiconductor materials. These are generally metal oxides or sulfides in the form of fine particles that consequently have the additional advantage of easily being removed by filtration or centrifugation. Product isolation is therefore easier and contamination by metals or other catalyst residues, that can be a problem with homogeneous PRCs, is avoided. Electrons in solid semiconductors occupy full valence energy bands (VB) that are separated from empty higher energy conduction bands (CB) by a band gap (BG) that is characteristic of the material. Upon photoexcitation of a semiconductor particle by light of energy greater than or equal to that of the band gap, an electron is promoted from the VB to the CB, leaving behind a positively charged hole. Electron/hole pairs (e^–^/h^+^) are thereby created. Holes are unoccupied states in the VB and can move in the particle as neighboring electrons shift to fill the hole. Thus the h^+^ behave as if they were positively charged particles. Upon separation, the e^–^ and h^+^ can migrate to the surface of the semiconductor particle where the electron can reduce an electron acceptor with a suitable redox potential to a radical anion (A^–•^) and/or the hole can oxidize an electron donor to the radical cation (D^+•^, Figure 1). Recombination, whereby the electron drops back down to the VB, occurs in competition with this in the bulk of the semiconductor particle or at the surface. Also possible is the back transfer of an electron from the acceptor/donor species to the semiconductor.

Many semiconductors are suitable as photoredox catalysts, ZnS (BG = 3.6 eV), CdS (BG = 2.4 eV), WO_3_ (BG = 2.8 eV) and Fe_2_O_3_ (BG = 2.3 eV) being popular choices. It is widely accepted that titanium dioxide (TiO_2_) in its various mineral forms is by far the most effective and reliable. This is down to its harmlessness, low cost, high activity and stability under irradiation. TiO_2_ has a large band gap which lies in the UVA region of the spectrum (anatase excitation wavelength 385 nm, band gap 3.2 eV). Whilst this is less convenient than visible light, the band edge positions are consequently more powerful redox agents. Of the naturally occurring crystal structures of TiO_2_, anatase is superior to rutile for photocatalytic activity [12]. For semiconductor photocatalyzed reactions Aeroxide (Evonik, formerly Degussa) P25 is the most widely employed form of TiO_2_ and is considered the “gold standard”. It has a relatively large surface area (≈50 m^2^ g^−1^) and consists of roughly 75% anatase and 25% rutile [13]. This combination exhibits a synergistic effect and is much more active than anatase or rutile alone. It is thought that contact between the two phases enables the rutile to act as an electron sink, preventing recombination and increasing the photoactivity of the holes in the anatase. Alternatively, catalytic “hot spots” at the interface between the phases might be the reason for this synergy [14].

Experimental set-ups are comparatively simple. PRC reactions can be carried out in tubular Pyrex reaction vessels such as Schlenk tubes. In C–C bond-forming processes the apparatus, P25 catalyst, solvents and reactants must be dried and purged with inert gas to remove oxygen. Stirring is maintained during photolysis to keep the TiO_2_ particles evenly dispersed. With P25 catalyst, irradiation with two hemispherical banks of 15 W Cleo tubes (λ = 350 nm) (for example two face tanning units) is maintained at ambient temperature. For some surface-modified TiO_2_ catalysts visible light can be used [15–16]. Following this the P25 can be removed by filtration through a Celite pad, or by centrifugation, and can usually be reused. Alternatively, glass beads coated with TiO_2_ can be used but these tend to rise to the solvent surface thus making irradiation less efficient. Tubes with thin internal coatings of TiO_2_ have also been employed with some success (see below); research to improve their reusability is underway.

## Review

### Generation of ROS and organic oxidations

**Figure 2:**
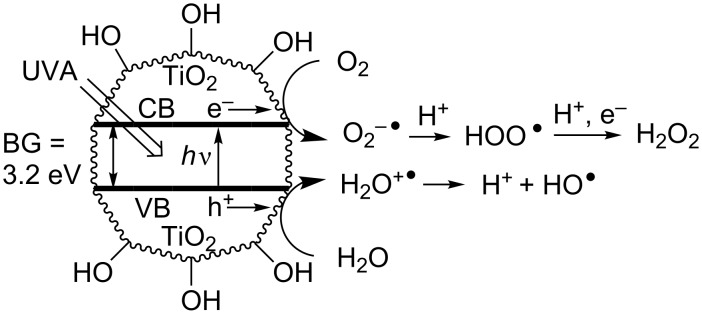
Photoredox activity of TiO_2_ with moist air.

When TiO_2_ is irradiated in the presence of moisture and atmospheric oxygen, the water acts as the donor and ‘picks up’ holes thereby releasing a cascade of extremely reactive hydroxyl radicals (see Figure 2).

Oxygen acts as the acceptor, picks up electrons from the semiconductor surface, so yielding superoxide anions that are converted to hydroperoxyl radicals and to hydrogen peroxide on successive reductions and protonations. The ROS generated in these ways oxidize any organic components or pollutants, by well-known oxidation and autoxidation processes, eventually leading to complete mineralization with production of CO_2_, water and mineral acids. Research in this area has been extensive [17] and as a result many different classes of organic (and inorganic) pollutants have been shown to undergo photodegradation. Degradative applications of TiO_2_ semiconductor photoredox catalysis (SCPC) have become important measures for environmental remediation. Organic and inorganic pollutants present in water, soil, and the atmosphere can be completely mineralized by this means. Furthermore, self-cleaning glass [18], bathroom and kitchen tiles, thinly coated with TiO_2_, are supplied by several manufacturers. As sunlight falls on the ceramics the oxidative process is promoted and the material stays clean. Another application undergoing extensive trials is of self-cleaning garments [19] that may prove effective particularly for army wear in desert conditions.

**Scheme 1:**
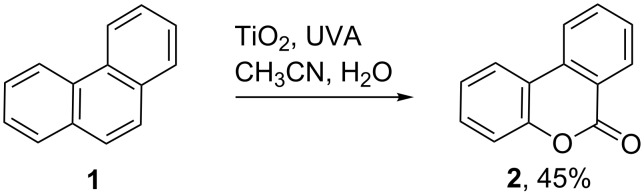
TiO_2_ promoted oxidation of phenanthrene [29].

ROS generated from semiconductor photoredox catalysis (SCPC) have also been put to use in a good number of preparative oxidations so taking the place of toxic metals (such as chromium and manganese) [20–22]. Many SCPC oxidations of aromatics have been reported, but of these it is perhaps the oxidation of benzene to phenol that is the most industrially significant [23–24]. Unfortunately, product selectivity is poor because the phenol itself undergoes further oxidation. SCPC oxidations that take place with varying success include toluene to benzaldehyde and benzoic acid [25], cyclohexane to cylohexanone [26], benzylic and allylic alcohols to carbonyl compounds [27] and alkene epoxidations [28]. Conventional syntheses of coumarins, which are important constituents of pharmaceuticals and dyestuffs, are non-trivial and involve multiple steps. Consequently, the one-pot SCPC oxidation of phenanthrene (**1**) to benzocoumarin (**2**), with a dispersion of TiO_2_ in aqueous, oxygenated CH_3_CN [29], is very appealing from a green chemistry point of view (Scheme 1).

### First discoveries of SCPC mediated bond forming processes

Several investigations during the 80s and early 90s demonstrated that bond forming organic transformations could be accomplished using SCPC [30–34], but these were mainly spectroscopic and/or mechanistic studies on the formation of well-known compounds. Notably, Bard and co-workers investigated the photo-Kolbé reaction, in which simple carboxylic acids RCO_2_H were decarboxylatively reduced to the corresponding alkanes RH on irradiation with platinized TiO_2_ in CH_3_CN under anaerobic conditions [35–37]. Only adamantane, from adamantane-1-carboxylic acid, was isolated and characterized. That the derived C-centered radicals R^•^ were intermediates was implied by EPR detection of Ph_3_C^•^ (from Ph_3_CCO_2_H) and the PBN spin adduct of CH_3_^•^ radicals from acetic acid [38].

**Scheme 2:**
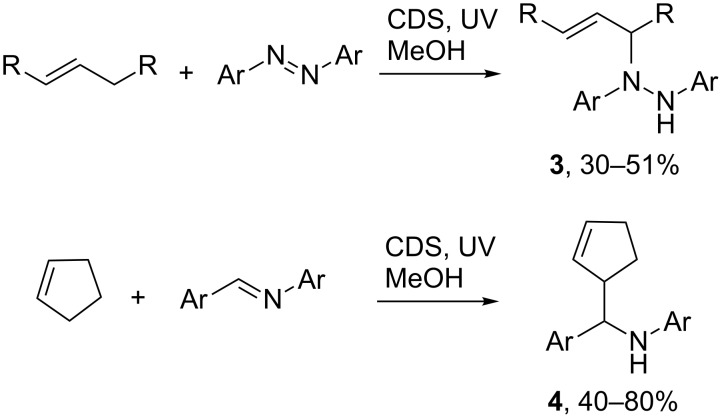
SCPC assisted additions of allylic compounds to diazines and imines [40–42].

The first successful applications of SCPC to the formation of novel organic compounds, that could be isolated on a gram scale, came from the research group of Kisch [39]. Additions of allylic alkenes and enol ethers to 1,2-diazines, mediated by photolyses of methanolic suspensions of CdS, resulted in the formation of alkenyl diazanes **3** (Scheme 2) [40]. Oxidation of the alkene/enol ether component by h^+^ from the CdS furnished radical cations. On deprotonation the derived allylic radicals added to the N=N bonds of the diazines resulting in modest yields of substituted diazanes **3**.

Functionalized *N*-benzylanilines **4** were also obtained from an analogous process in which alkenes added to imines (Scheme 2) [41–42]. The mechanism was believed to involve e^–^ capture by the imine thereby forming α-aminodiphenylmethyl radicals that underwent hetero-coupling with the allyl radicals generated from the allylic C–H containing substrates. Cyclohexanes, dihydrofurans, cyclopentenes and α-pinene were all compatible with this protocol, with yields ranging from 40–80% being recorded. The simultaneous activation of both reactants by reductive and/or oxidative interactions with the photoexcited semiconductor marked these processes as both elegant and efficient.

The foregoing examples have demonstrated the dual nature of the SCPC process that endows it with notable substrate flexibility. Most commonly a well-designed donor precursor is employed that picks up h^+^ from the photoactivated semiconductor to afford radical cations that subsequently transform by H^+^ loss (or otherwise) to neutral radicals. A sacrificial electron acceptor is required to scavenge the electrons from the e^−^/h^+^ pair. This may be a solvent, excess of an electron-deficient substrate or an additive. The neutral radicals can then be exploited in addition, cyclization, coupling or reductive processes. The alternative process employs an acceptor precursor with a suitable redox potential to pick up e^–^ from the semiconductor with production of a radical anion capable of transforming to a neutral radical. In this case a sacrificial donor is needed to scavenge the holes (usually an alcohol or an amine). Occasionally, as in some of the Kisch systems mentioned above, both h^+^ and e^–^ play complementary parts in a single preparative process. In the following sections the most recent developments will be reviewed; first with initial oxidative reactions and second with initial reductive processes.

### Initial oxidative processes with donor precursors

**Scheme 3:**
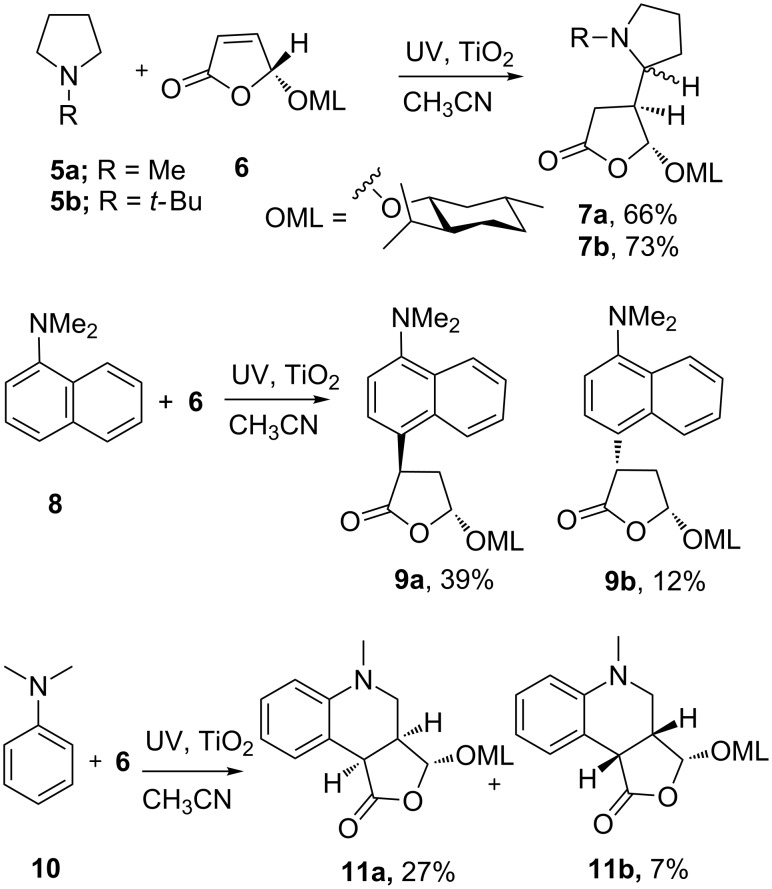
TiO_2_ promoted addition and addition–cyclization reactions of *tert*-amines with electron-deficient alkenes [43–47].

Hoffmann and co-workers demonstrated that tertiary amines N(CH_2_R)_3_ were suitable donors, transferring their *N*-lone pair electrons to photoexcited TiO_2_, CdS or ZnS thus generating aminium radical cations ^+•^N(CH_2_R)_3_. These deprotonated at an adjacent C-atom to furnish α-aminoalkyl radicals (RCH_2_)_2_NC^•^HR that added to electron deficient alkenes so facilitating several C–C bond forming and ring closing reactions [43–44]. *N*-Methyl- and *N*-*tert*-butylpyrrolidine (**5a** and **5b**) both added to the α,β-unsaturated menthyloxyfuranone **6** producing of pyrrolidinyldihydrofuranones **7** in good yields. The menthyloxy chiral auxiliary directed the addition of the amine moieties exclusively from the less hindered face but, due to poor selectivity at the chiral centers forming alpha to the nitrogen, the diastereomeric excesses of **7a**,**b** were poor (Scheme 3). The method was generalized for the addition of *tert-*amines and *N*-protected secondary amines to electron deficient alkenes. *N,N*-Dimethylnaphthalen-1-amine (**8**) also added to **6** in a TiO_2_ promoted SCPC process that produced two diastereomeric adducts of 4-(dimethylamino)naphthalen-5-menthyloxydihydrofuranone **9a**,**b** (51%) [45]. In this case the mechanism probably involved the coupling of the initial aminium radical cation with the radical anion formed by SET to the furanone substrate [46].

An interesting further convolution was observed in the TiO_2_ and ZnS promoted SCPC reactions of *N,N*-dimethylaniline (**10**) with furanone **6**. A tandem radical addition–cyclization to afford diastereomeric tetrahydrofuroquinolinones **11a,b** took place (Scheme 3) [47]. In the proposed mechanism, the photoexcited semiconductor oxidized **10** to a radical cation, which deprotonated to give the nucleophilic *N*-methylanilinomethyl radical that added to the double bond of **6**. The major product **11a** was formed by attack of this species *anti* to the menthyloxy substituent of **6**. The electrophilic intermediate radical thus formed readily ring closed on to the electron rich aromatic ring furnishing **11a** and **11b** after oxidative rearomatization.

**Scheme 4:**
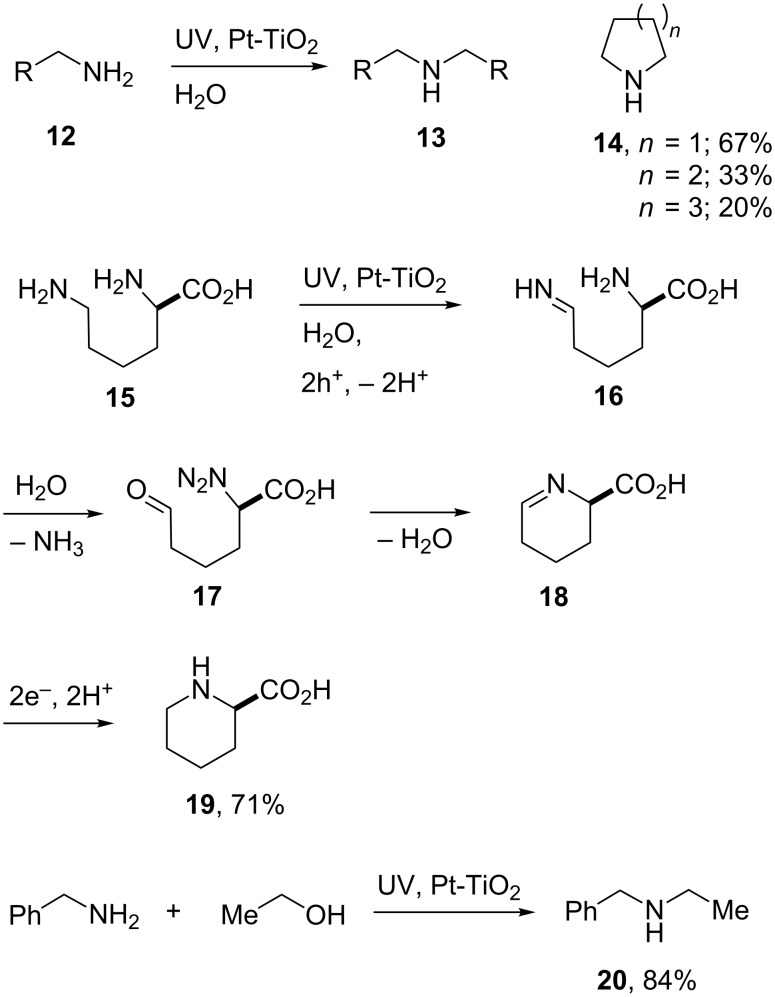
Reactions of amines promoted by Pt-TiO_2_ [48–49].

When primary amines **12** were irradiated on their own in an aqueous dispersion of a Pt-TiO_2_ catalyst, an interesting self-reaction occurred. Ohtani and co-workers isolated modest yields of secondary amines **13** that were formed via C–N coupling (Scheme 4, top) [48]. Terminal diamines reacted by ring closure resulting in cyclic amines **14**; pyrrolidine was isolated in a good yield but the process was less effective with 6- and 7-membered rings (Scheme 4).

When the water in these amine reactions was replaced by alcohols, photolyses with Pt-TiO_2_ led to coupling and the formation of the corresponding secondary or tertiary amines; for example **20** (Scheme 4) [49]. Two successive SET’s from the alcohol to the photoexcited TiO_2_ catalyst, and subsequent proton loss, brought about conversion to the corresponding aldehyde. Imine condensation with the amine ensued, followed by reduction–protonation yielding the coupled product **20**. The reaction occurred readily in the case of benzylic amines and piperidine, but aliphatic amines and aniline exhibited poor reactivity.

Carboxylic acids are available in great variety from natural and industrial sources. Green synthetic methods based around them as starting materials are undoubtedly worthy of development. That simple carboxylic acids RCO_2_H release radicals (R^•^) in the photo-Kolbé reaction was referred to above. Ni and co-workers showed that vinyl acetate polymerization could be mediated by TiO_2_ nanoparticle photocatalyzed reactions with carboxylic acids [50]. Kinetic studies established that amongst simple carboxylic acids, in aqueous solutions, *n*-butyric acid displayed the highest initiation rate.

**Scheme 5:**
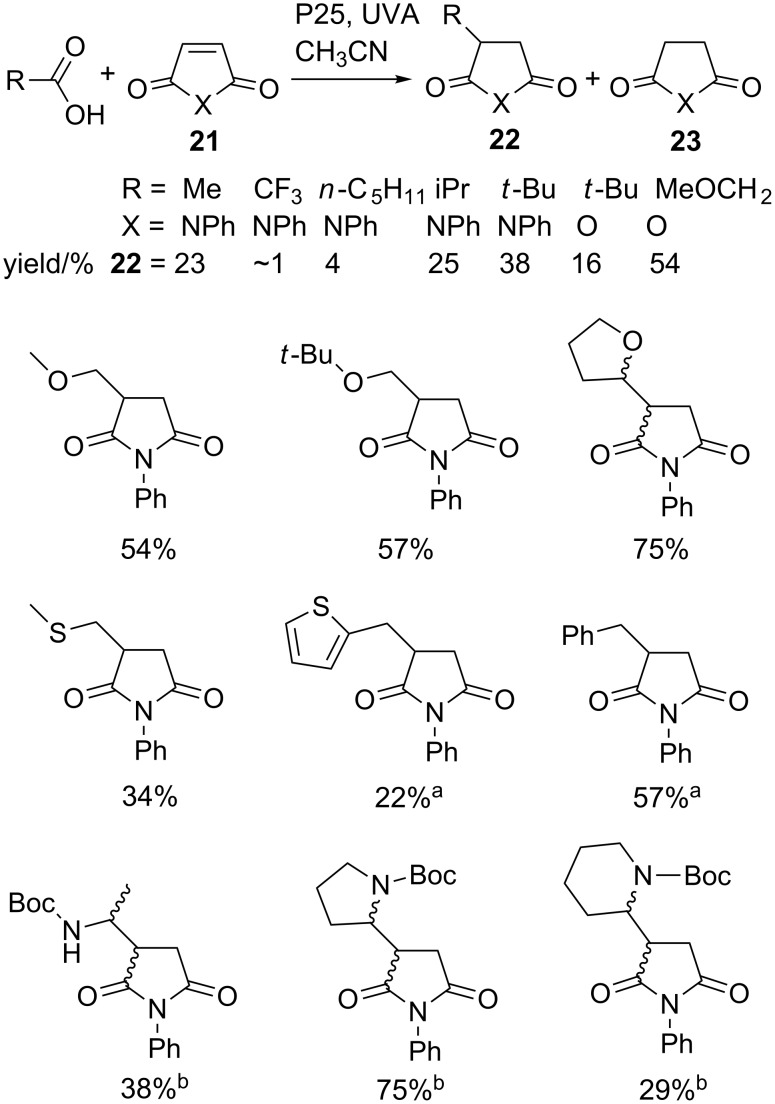
P25 Promoted alkylations of *N*-phenylmaleimide with diverse carboxylic acids [53–54]. ^a^Accompanied by R–R dimers. ^b^Obtained as 1:1 mixtures of two diastereoisomers.

Some research using carboxylic acids and employing homogeneous PRCs has been reported [51–52]. However, the precedent of the photo-Kolbé reactions implied that radicals generated from carboxylic acids by semiconductor methods could be enlisted in constructive molecular assembly applications. Our study of SCPC processes revealed that a wide range of carboxylic acids could be deployed in preparatively useful ways. Radical additions to alkenes, cyclizations and tandem addition–cyclizations, as well as homo-couplings, were demonstrated. P25 promoted UVA photolyses of aliphatic carboxylic acids RCO_2_H with electron-deficient alkenes (Z) in dry CH_3_CN under anaerobic conditions led to the formation of adducts RZH incorporating the carboxylic acid moiety R and an additional H atom [53–54]. Yields were low for *n*-alkyl radicals, but a trend towards higher yields for branched acids and radicals with higher stabilization energies was observed (Scheme 5). In each case significant amounts of the reduced alkene (HZH, **23**) accompanied adducts **22**; indicating that excess alkene acted as the sacrificial e^–^ acceptor. A sample of the diverse functionalized carboxylic acids that take part in decarboxylative additions to maleimides is presented in Scheme 5.

Reasonable yields of adducts were obtained for alkyl radicals with α-alkoxy substituents. Two diastereomers of **22** as a 1:1 mixture were obtained from 2-tetrahydrofuroic acid in a very pleasing yield of 75%. Phenyl- and 2-thienylacetic acids afforded adducts accompanied by the dimers bibenzyl (18%) and 1,2-di(thiophen-2-yl)ethane (27%), respectively. Significantly, Boc-protected α-amino acids also proved amenable and yielded adducts as 1:1 mixtures of stereoisomers. Chirality was not preserved with optically pure amino acids probably because the released radicals were planar, or close to planar. Radical additions took place to other alkene substrates such as acrylamide but extensive polymerization was observed with methyl methacrylate.

The phenoxyacetic acid/acrylamide reaction was chosen to test different forms of the TiO_2_ catalyst. The yield of the 4-phenoxybutanamide adduct was slightly less with Millennium PC500 [55], which is mainly anatase and has about six times the surface area of P25. SCPC reactions in NMR tubes coated internally with TiO_2_ by a sol–gel process [56] gave the best yields, but the coatings detached after about three usages. Lower conversions and yields resulted when photospheres, comprising hollow Pyrex beads coated with TiO_2_ [57], were employed; probably because of the difficulty in dispersing them evenly during photolysis. The standard P25 catalyst was re-affirmed as the best compromise.

**Scheme 6:**
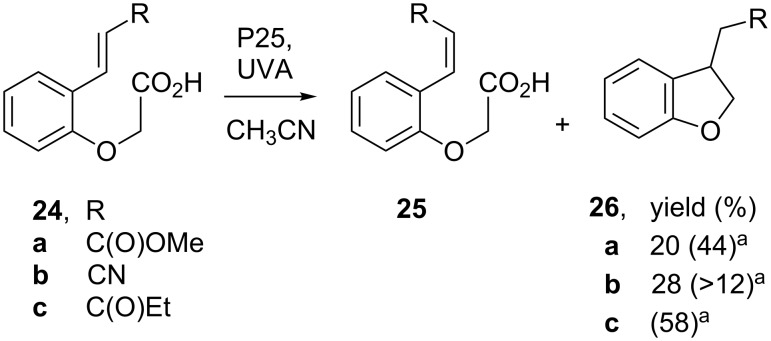
SCPC cyclizations of aryloxyacetic acids with suitably sited alkene acceptors [54]. ^a^Yields in brackets are maximum yields from NMR monitoring of reactions in sol–gel TiO_2_ coated tubes.

Reactions of aryloxyacetic acids functionalized with suitably sited alkene **24**, aromatic or oxime ether acceptors, were carried out with P25 under standard conditions and also in sol–gel TiO_2_ coated NMR tubes (see Scheme 6). For styrenyl acids **24** control photolyses in the absence of TiO_2_ showed that *E/Z* isomerisation was significant (**25**, 52–79%). Rather complex product mixtures containing *E* and *Z*-isomers of unreacted alkene, as well as low yields of dihydrobenzofurans **26**, were obtained from **24a–c**. Poor results were obtained from acids with aromatic and oxime acceptor substituents.

**Scheme 7:**
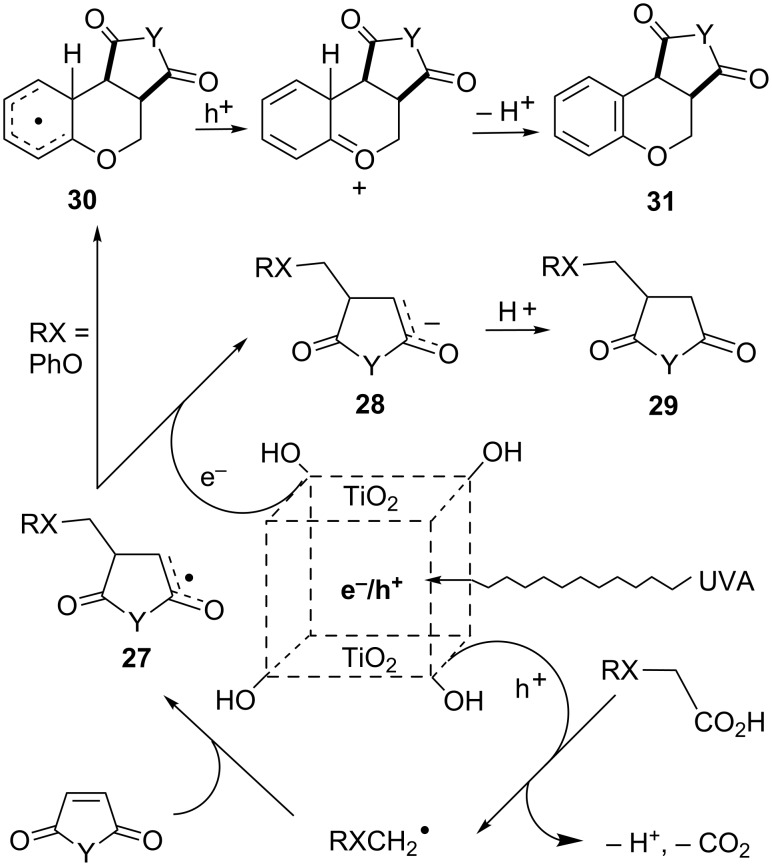
TiO_2_ promoted reactions of aryloxyacetic acids with maleic anhydride and maleimides [53–54].

The P25 SCPC reactions of aryloxyacetic acids with maleic anhydride or maleimide substrates gave the expected adducts **29** but also launched an alternative reaction channel resulting in novel chromenedione derivatives **31** (Y = O, NR). These are evidently formed when the initial adduct radicals **27** cyclized onto the aryl rings **30** followed by rearomatization (Scheme 7). Good to excellent overall yields were obtained under all conditions. Excess maleic anhydride or maleimide was necessary as the sacrificial electron sink; hence moderate amounts of succinic anhydride or succinimides were byproducts. The standard reactions with just P25 gave a slight excess of the chromenopyrrole for most substituents, but the selectivity could be tuned to some extent by modifications to the catalyst. Generally, chromenopyrrole **31** (Y = NR) production was favored by using a more dense dispersion of P25. In contrast, selectivity was reversed with predominant formation of adducts **29**, when the sol–gel TiO_2_-coated tubes were employed.

Dissociation of carboxylic acids to carboxylate and a proton occurs at the TiO_2_ surface [58]. The TiO_2_ surface is dotted with hydroxy groups [59–60], and it is probable that these trap the valence band holes [61]. Electron transfer from the carboxylate π-orbitals to a hole trap site supplies surface bound RXCH_2_-CO_2_^•^ radicals. Rapid β-scissions of these species yield the desired free RXCH_2_^•^ radicals. That these species are intermediates has been confirmed by detection of their EPR spectra in both solution [38,54–55], and solid state studies [62]. This decarboxylation is favorable for precursors that generate stabilized RXCH_2_^•^ radicals, thus explaining the superior yields and conversions recorded. Radical stabilization from the simple aliphatic acids of Scheme 5 (top) is minimal; so back transfer of an electron to the TiO_2_ will compete with the loss of CO_2_, thus reducing yields.

An intriguing aspect of the process is the source of the H atoms gained during the formation of adducts **22** and **29**. This was not the proton lost from the aromatic ring in forming **31**. Deuterium labelling experiments established that neither the solvent nor the protons of the carboxylic acid groups were the H atom sources. It is well established that HO groups are attached to the surface of the TiO_2_ particles [44], and these most likely supply the additional H atoms. It is evident that the P25 acts as both a catalyst and a reaction partner because on photoactivation it supplies electrons and holes while also donating protons from surface hydroxy groups.

An interesting adjunct to these results was our finding that aryloxy-, arylthio- and anilino-acetic acids, carrying electron donor substituents (2-, 3- or 4-MeO, *t*-Bu, MeS or di- and tri-MeO), underwent addition–cyclization reactions with maleimide on UVA irradiation, but in the complete absence of photoredox catalysts [63]. Good to excellent yields of chromenopyrrolediones and pyrroloquinolinediones (**32**, X = O, NR) were obtained from photolyses in H_2_O/MeCN solutions; yields of thiochromenopyrrolediones (**32**, X = S) were poor due to competing reactions. No direct adducts (analogues of **29**) were formed as byproducts in these reactions.

**Scheme 8:**
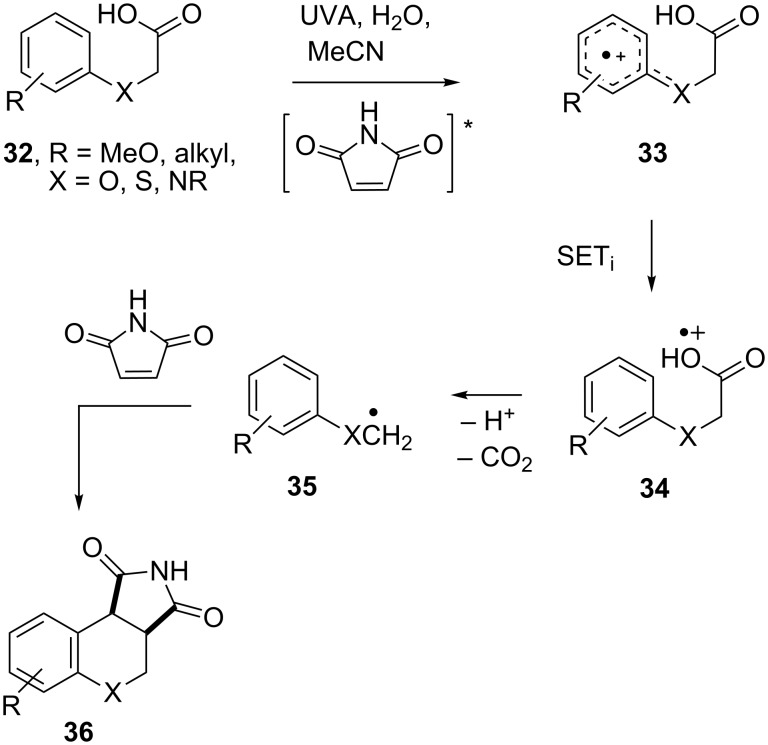
Photoredox addition–cyclization reactions of aryloxyacetic and related acids promoted by maleimide [63].

Radical cations **33** were generated by SET from the electron-rich acids to the photoexcited maleimide and then underwent internal SET_i_ with production of radical cations **34** (Scheme 8). The latter deprotonated and lost CO_2_ thus supplying radicals **35** that took part in addition–cyclization reactions with maleimide producing **36** in the same way as described above in the TiO_2_ mediated process.

Homo-dimers are an important motif in many natural products and pharmaceuticals. Transition metals are very successful in catalyzing homo-couplings, particularly for sp^2^ hybridized C-centers. Radical-based dimerizations are also known to be effective and offer the opportunity to extend the scope to sp^3^ centers. TiO_2_ photoredox reactions with carboxylic acids generate neutral C-centered radicals and therefore offer the opportunity to reap the benefits of clean SCPC methodology in homo-coupling processes. We found that homo-dimers **39** were indeed formed from UVA photolyses of carboxylic acids in MeCN containing dispersions of P25 [54,64]. The most efficient system employed 8 equivalents (with respect to the acid) of P25 doped with 0.1% (w/w) of Pt metal [65]. The released radicals **37** either dimerized to **39** or picked up e^−^ and H^+^ from the TiO_2_ surface thus yielding the reduction products **38**; as in the photo-Kolbé process. With our methodology homo-dimers were obtainable from most carboxylic acids but best yields resulted when the released radicals were stabilized by functional groups, particularly benzyl and related moieties.

Benzyl type radicals were the most efficient and selective (**39**, R^1^ = R^2^ = H, 87%) with no formation of the reduction product (toluene, **38**). Diphenylacetic acid yielded tetraphenylethane in a near-quantitative yield but in contrast, the related 9*H*-fluorene-9-carboxylic acid produced the reduced alkane (9*H*-fluorene) as the major product. Incorporation of substituents on the phenyl ring proved a success; arylacetic acids bearing both electron releasing and electron withdrawing groups provided coupled dimers in high yields. Even the extremely electron poor perfluorophenylacetic acid afforded 53% of the dimer. Naphthalenylacetic acids furnished dimers but accompanied by significant quantities of reduction product **38**. Heteroaromatics provided the desired dimers in pleasing yields. With α-substituted arylacetic acids (R^1^ and/or R^2^) the outcome depended critically on the type of substituent. Racemic α-fluorophenylacetic acid was converted to two stereoisomeric dimers **39** (R^1^ = F, R^2^ = H) in an excellent combined yield of 90%. Remarkably, incorporation of a second fluorine substituent at the same position in α,α-difluorophenylacetic acid essentially shut down the reaction. SCPC of acids with unprotected R^1^ = OH or NH_2_ followed other reaction channels.

**Scheme 9:**
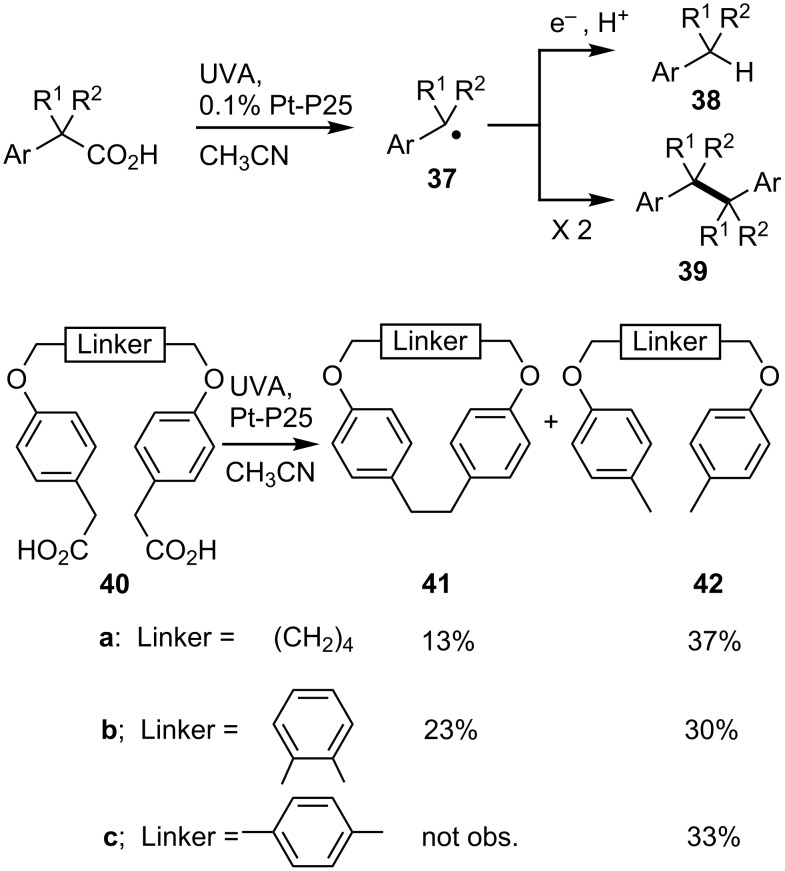
SCPC promoted homo-couplings and macrocyclizations with carboxylic acids [64].

SCPC of dicarboxylic acids with suitably designed architectures seemed to offer a novel protocol for macrocyclizations. In view of the generally high dimer yields obtained from arylacetic acids, we investigated SCPC reactions of the set of precursor diacids containing two of these units linked in different ways (**40a–c**, Scheme 9). The SCPC reaction of **40a**, with a flexible methylene linker, gave doubly reduced **42a** as the major product. Pleasingly, however, the dioxadibenzenacyclododecaphane **41a** was also obtained in a modest yield (13%). Diacids **40b** and **40c** were designed with rigid, planar phenyl ring linkers. TiO_2_ SCPC of the *ortho-*analogue **40b** was more successful with the cyclophane **41b** being formed in 23% yield along with 30% of **42b**. Diacid **40c** returned only the dialkane product **42c** probably because the two reaction centers were held too far apart by the rigid *p-*xylene linker.

**Scheme 10:**
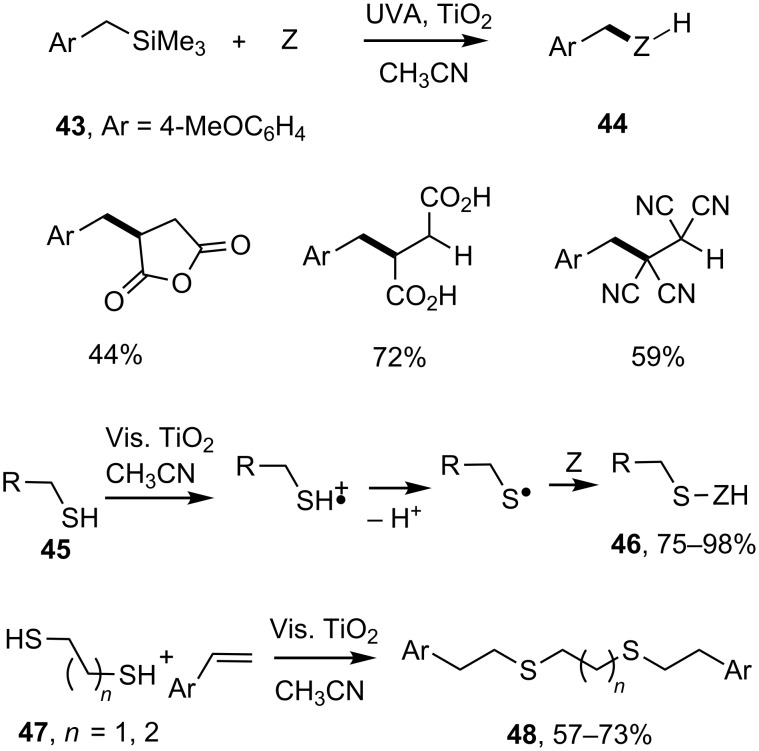
TiO_2_ promoted alkylations of alkenes with silanes [66] and thiols [67].

Some other compound types have proved to be effective donors in SCPC, picking up h^+^ from the semiconductor and releasing neutral radicals. Albini and coworkers reported on the use of benzyltrimethylsilanes **43** [66]. Hole oxidation with photoexcited dispersions of TiO_2_ gave rise to radical cations that underwent fragmentation with release of benzyl type radicals. The latter reductively benzylated electron-deficient alkenes; including maleic anhydride, maleic acid and related nitriles, in good yields (Scheme 10). Excess alkene was required to act as a trap for the conduction band electrons. In one instance a multigram reaction was successfully carried out with sunlight as the irradiation source.

Recently Greaney and co-workers discovered that thiols **45** were also suitable donors with photoactivated TiO_2_ [67]. The thiol radical cations, formed on hole capture by the thiols, lost protons and generated thiyl radicals (Scheme 10). Benzenethiol, thiophenols and *n*-alkylthiols all afforded reduced alkene adducts **46** in high yields, but steric hindrance in branched thiols was deleterious. Alkenes with electron-releasing and electron-withdrawing substituents were tolerated. Interestingly, SCPC reactions also took place such that ethane and propanedithiols **47** coupled with two styrene molecules affording 1,2-bis(arylethylthio)alkanes **48** in good yields (Scheme 10).

### Initial reductive processes with acceptor precursors

Photoexcited TiO_2_ is only weakly reducing (−0.3 eV) and consequently less attention has been given to its role in preparative SCPC with acceptor molecules. In order to carry out such reductive transformations an excess of a sacrificial electron donor (reductive quencher) is needed to scavenge the VB holes and prevent e^−^/h^+^ recombination. Alcohols and amines have been successfully deployed in this role.

**Scheme 11:**
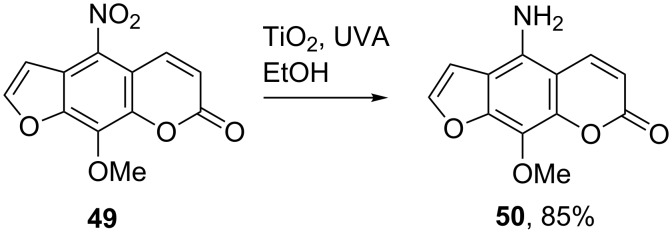
TiO_2_ reduction of a nitrochromenone derivative [70].

Reductions of nitroaromatics took place effectively in photoactivated aqueous TiO_2_ slurries, with methanol, ethanol or isopropanol as hole scavengers [68–69]. This method was successfully applied in the reduction of 5-nitro-8-methoxypsoralen (**49**) to 5-amino-8-methoxypsoralen (**50**) in an ethanolic dispersion of TiO_2_ (Scheme 11) [70]. Mechanistically, it was thought that the reaction proceeded via a series of two electron reductions.

**Scheme 12:**
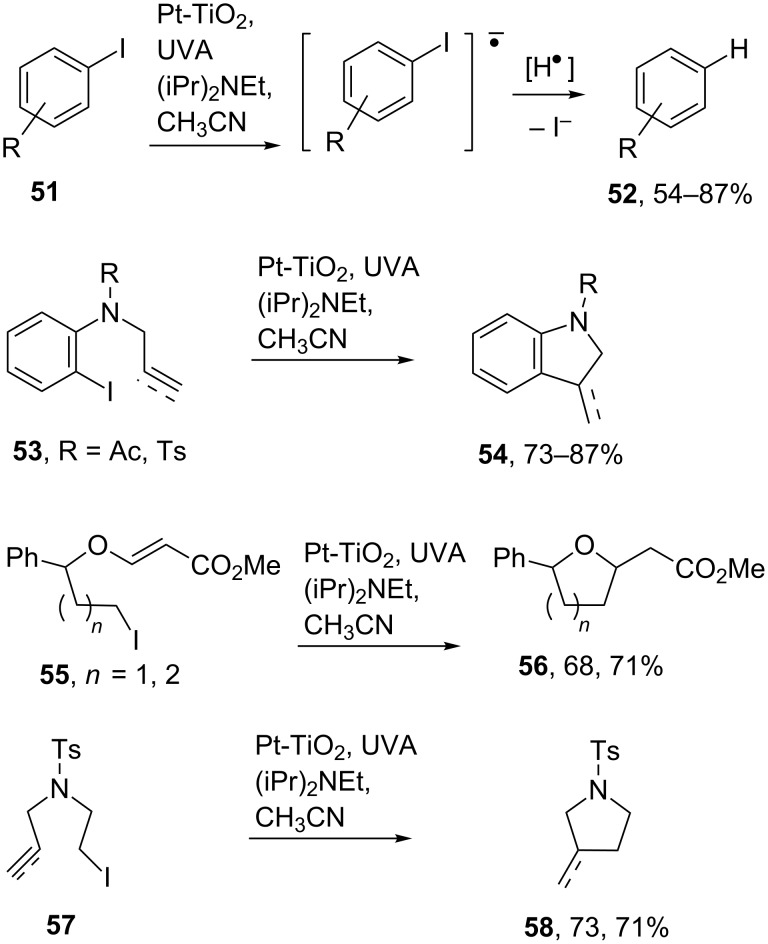
TiO_2_ mediated hydrodehalogenations and cyclizations of organic iodides [71].

The scope of reductive photoredox applications was significantly boosted by Scaiano and co-workers’ discovery that organic halides function as acceptors with platinized TiO_2_ nanoparticles [71]. They used (iPr)_2_NEt as the sacrificial donor and demonstrated that reductive hydrodehalogenations of both electron-rich and electron-poor aryl iodides **51** took place in good yields (Scheme 12). Furthermore, unactivated aryl **53** and alkyl iodides **55** and **57** bearing unsaturated side chains underwent reductive 5- and 6-*exo-*cyclization reactions enabling several types of heterocycles (**54**, **56**, and **58**) to be accessed.

Hydrogenations of alkenes with H_2_ gas employing TiO_2_ loaded with Pd (and other metal catalysts) is a standard procedure [72]. The reductions of CO_2_ by SCPC have received extensive attention [73–78], albeit mostly using the more strongly reducing semiconductors as catalysts.

**Scheme 13:**
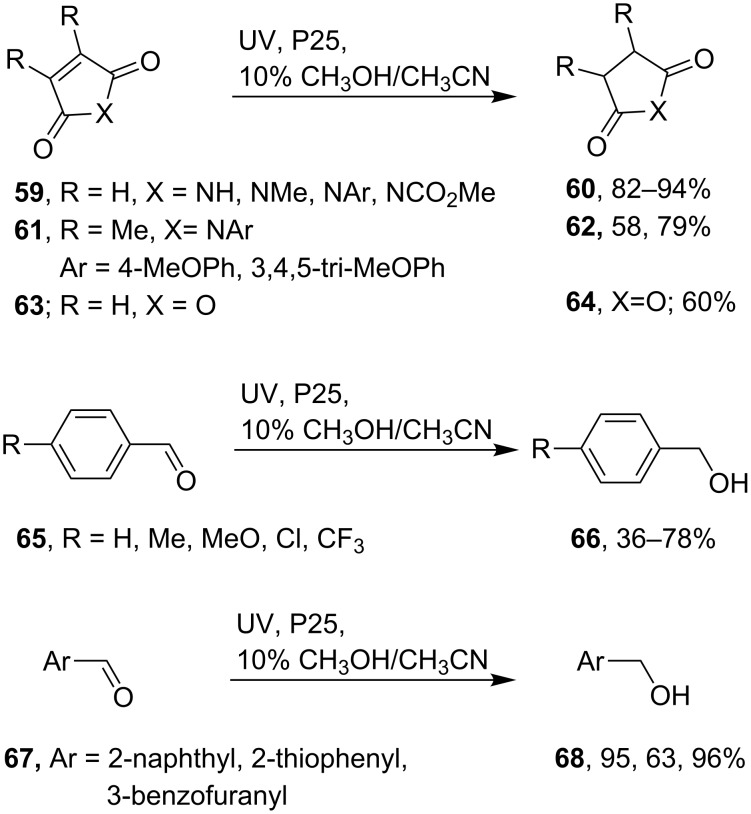
TiO_2_ promoted hydrogenations of maleimides, maleic anhydride and aromatic aldehydes [79].

We observed that during TiO_2_ promoted alkylations of maleimides with carboxylic acids, the products always included significant amounts of succinimides, so simple hydrogenation had occurred [53–54]. Evidently the maleimides operated as electron sinks in two sequential reduction–protonations, in addition to acting as the radical acceptors. This led to the idea that TiO_2_ based SCPC could be a mild way for hydrogenating electron deficient alkenes without the need to deploy hydrogen gas. In practice, when *N-*phenylmaleimide was irradiated with a dispersion of P25 in CH_3_CN containing 10% methanol as hole-scavenger *N-*phenylsuccinimide was indeed produced almost quantitatively. Good to excellent yields (82–94%) of the corresponding succinimides were obtained from a range of *N*-substituted maleimides (Scheme 13) [79]. Exceptions were starting maleimides with 3- and or 4-substituents on their C=C double bonds. Satisfactory yields were, however, obtained for 3,4-dimethyl-substrates as long as electron donating MeO substituents were present in the *N*-aryl rings (**61**); but not with an *N*-4-chloroaryl substituent. Hydrogenation of the highly electrophilic maleic anhydride **63** furnished succinic anhydride **64** but a significant quantity of ring opened byproduct was also formed.

Carbonyl compounds are also good electron acceptors and so SCPC hydrogenations seemed likely. Actually there is a literature precedent for photocatalytic hydrogenation of acetophenone derivatives with TiO_2_ [80–81]. We studied a representative range of aldehydes and ketones using dispersions of P25 with soft UVA in MeCN/MeOH (9:1) as solvent [79]. Aromatic aldehydes yielded the corresponding primary alcohols along with pinacol byproducts. Control experiments confirmed that these diols were formed by the well-known photochemical coupling process. We found, however, that diol contamination could be largely suppressed when denser dispersions of P25 (1 mg mL^−1^, ≈0.75 equiv) were employed resulting in the desired alcohols. Ketone hydrogenations were less successful. Aryl aldehydes with either electron-releasing or electron-withdrawing substituents, **65**, returned the corresponding benzyl alcohols **66** in moderate to good yields. Naphthaldehyde and heteroaromatic aldehydes **67** also hydrogenated very smoothly under these conditions (Scheme 13).

**Scheme 14:**
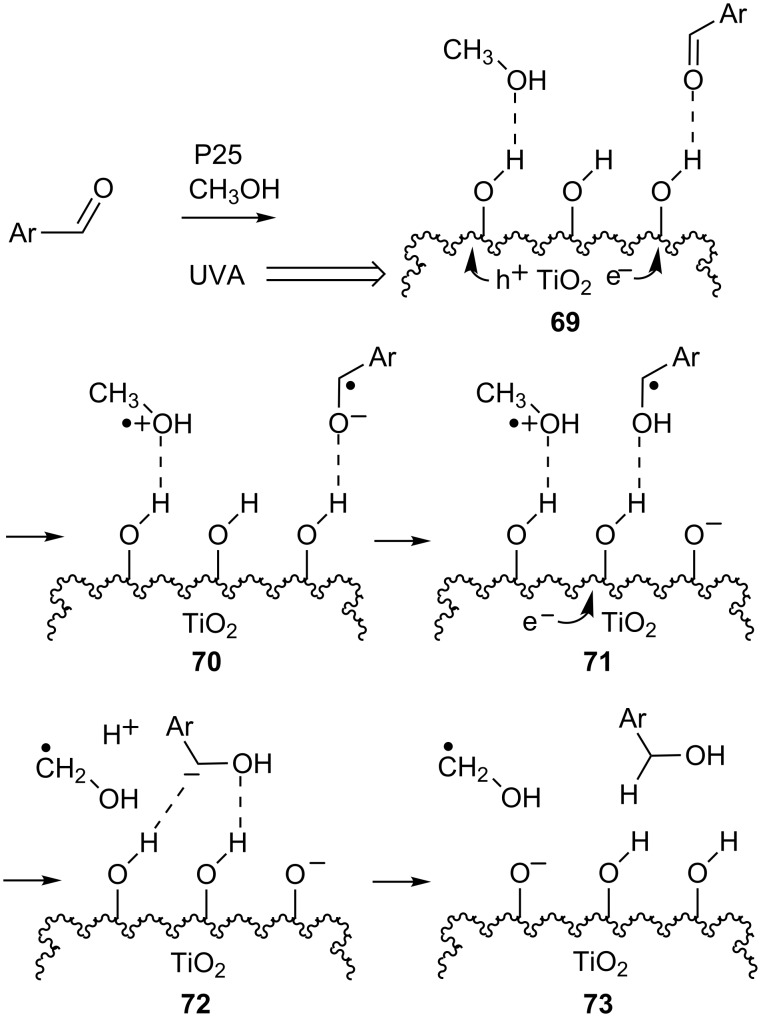
Mechanistic sketch of SCPC hydrogenation of aryl aldehydes.

The HO groups attached to the TiO_2_ surface probably again play key roles in TiO_2_ SCPC hydrogenations. The incoming aldehyde H bonds to a surface OH group (**69**), then readily accepts e^−^ from an e^−^/h^+^ pair (**70**, Scheme 14). The solvent methanol also H bonds to surface OH groups where it scavenges h^+^ and also exchanges protons with surface OH groups as the radical cation is generated (**70**). The aryl radical anion in **70** picks up a proton from the surface so producing the resonance stabilized hydroxybenzyl radical in **71**. The latter is reduced to the benzyl anion by a second e^−^ from the TiO_2_ and is then protonated by a neighboring surface OH (**72**). The benzyl alcohol product is released most likely along with a hydroxymethyl radical. Product deuteriation in an experiment with CD_3_OD in place of CH_3_OH supported this mechanism.

## Conclusion

The experimental convenience, low cost of the semiconductor and the ease of product isolation ensure increasing popularity of preparative SCPC methods. There is a clear opportunity for surface modification of the semiconductors with metals or dyes to extend the capability into the visible light region. This is a step nearer to directly channeling the Sun’s energy into synthetic chemistry. Though not proven beyond doubt, the likelihood is that surface OH groups on the semiconductor play important mechanistic roles. It is counterintuitive that metal oxides act as reducing agents, but the plentiful surface OH groups are probably the source of the H atoms supplied in reductive SCPC alkylations and hydrogenations. In those cases the semiconductor is really a reaction partner as well as a catalyst so not surprisingly, the density of its dispersion is an important experimental parameter. SCPC offers a benign way of making radicals, free from toxic organotin compounds, or pyrophoric initiators, and so adds to the growing portfolio of such methods [82–85]. Compounds so far accessed include adducts from alkenes and imines, a wide range of heterocycles from ring closures, radical homo-dimers and macrocycles as well as hydrogenated products from nitro-aromatics, alkenes and aldehydes. There is obvious scope for the discovery of additional donor and acceptor precursors. For example, it was recently shown that acyloximes act as hole acceptors with homogeneous PRCs [86] so it is likely that these, and related oxime derivatives [87], will also function with semiconductor catalysts. Similarly, α-keto acids are readily available and are promising candidates for SCPC generation of acyl radicals. Radical polymerizations are hugely important industrially and it seems certain SCPC has a role to play in this area too.
